# Maternal mortality by socio-demographic characteristics and cause of death in South Africa: 2007–2015

**DOI:** 10.1186/s12889-020-8179-x

**Published:** 2020-02-01

**Authors:** Nolunkcwe J. Bomela

**Affiliations:** Department of Research Capacity Development, Nelson Mandela University, University Way, Summerstrand, Port Elizabeth, Eastern Cape 6031 South Africa

**Keywords:** Socio-demographic, Maternal mortality, South Africa

## Abstract

**Background:**

South Africa’s maternal mortality ratio remains high although it has substantially declined in the past few years. Numerous studies undertaken in South Africa on maternal mortality have not paid much attention to how the causes are distributed in different socio-demographic groups. This study assesses and analyses the causes of maternal mortality according to sociodemographic factors in South Africa.

**Methods:**

The causes of maternal deaths were assessed with respect to age, province, place of death, occupation, education and marital status. Data were obtained from the vital registration database of Statistics South Africa. About 14,892 maternal deaths of women from 9 to 55 years of age were analysed using frequency tables, cross-tabulations and logistic regression. Maternal mortality ratio (MMR), by year, age group, and province for the years 2007–2015 was calculated.

**Results:**

The 2007–2015 MMR was 139.3 deaths per 100,000 live births (10,687,687 total live births). The year 2009 had the highest MMR during this period. Specific province MMR for three triennia (2007–2009; 2010–2012; 2013–2015) shows that the Free State province had the highest MMR (297.9/100000 live births; 214.6/100000 live births; 159/100000 live births) throughout this period. MMR increased with age. Although the contribution of the direct causes of death (10603) was more than double the contribution of indirect causes (4289) maternal mortality showed a steady decline during this period.

**Conclusions:**

The study shows evidence of variations in the causes of death among different socio-demographic subgroups. These variations indicate that more attention has to be given to the role played by socio-demographic factors in maternal mortality.

## Background

Despite a 44% decline in maternal mortality worldwide from 1990 to 2015, South Africa’s maternal mortality remains high [1, 2]. Notwithstanding the high levels, South Africa has reported a decline in the number of maternal deaths^1^ and institutional Maternal Mortality Ratio (iMMR) since 2009 [2]. A notable decline in iMMR from a peak of 189 deaths per 100,000 live births in 2009 to 135 deaths per 100,000 live births was reported in 2016 [2, 3]. The Rapid Mortality Surveillance (RMS) of 2016, also noted the reduction in iMMR when they compared estimates from other sources [4]. The reduction in deaths from non-pregnancy related infections and the success of the Human Immunodeficiency Virus (HIV) antiviral treatment programme in pregnancy and beyond have been hailed as the main reason for the decline in iMMR in South Africa during the past few years [2]. An inventory of progress made on the Sustainable Development Goals (SDGs) in South Africa revealed significant advances made in development, including health, over the past 25 years, as well as the considerable challenges that remain. This report, in reference to Goal 3 of the SDGs which is to *ensure healthy lives for all and promote well-being for all at all ages*, states that improvements in health services in the country have reduced maternal and child mortality rates and the incidence of some communicable diseases [1, 5]..

Since its inception in 1997, the National Committee on Confidential Enquiries into Maternal Deaths (NCCEMD) which records and analyses all institutional maternal deaths (maternal deaths that occur outside the state facilities are excluded in these records and analyses) has published seven reports on maternal deaths in South Africa. These reports describe at length the magnitude of the problem of maternal deaths, the pattern of disease causing maternal deaths, the avoidable factors, missed opportunities and substandard care related to these deaths. Thereafter, they make recommendations to health officials and the national Department of Health regarding approaches to be employed towards reducing the number of maternal deaths [2, 6–11]. Moreover, the reporting of maternal deaths has vastly improved and has become more efficient and reliable leading to early identification of problems such as preventable deaths, poor clinical assessment, delays in referral, poor monitoring of patients and lack of appropriately trained doctors. Notwithstanding these successes there is still much to do to improve and reduce maternal mortality ratio in South Africa to acceptable levels.

The most recent NCCEMD report published in January 2018 notes that all provinces, except Limpopo Province (LP) have shown a decline in maternal deaths in recent years. According to this report non-pregnancy related infections remained the largest category of maternal deaths between 2002 and 2013, however, a significant drop in these infections has been recorded [2]. A noticeable drop in a number of maternal deaths due to direct causes especially hypertension and pregnancy-related sepsis was recorded while there was an increase in deaths due to obstetric haemorrhage, ectopic pregnancies and abortion during the 2002–2013 period [2]. Moreover, the NCCEMD affirms that great strides have been made by the government in reducing maternal deaths in South Africa. However, the influence of socio-demographic factors on maternal deaths is less well-explored by both the NCCEMD and the RMS which regularly publish reports at national level on the status of maternal mortality in South Africa.

To date, a very limited number of studies have been undertaken in South Africa to analyze the contribution of socio-demographic factors on maternal mortality [12–14]. This paper extends the previous analysis of maternal mortality between 2002 and 2006 by this author [14] by using data collected by the Department of Home Affairs (DHA) and subsequently analysed by Statistics South Africa (StatsSA). Many studies assess determinants of maternal mortality in a fragmented scope using a few cases of maternal deaths without acknowledging the impact of socio-demographic factors on maternal mortality. This study assessed the contribution of these factors on 14,892 maternal deaths that were recorded over a period of 9 years (2007–2015). Studies on maternal mortality in Ghana and United States found evidence of variations in the causes of maternal mortality among different sociodemographic subgroups [15, 16].

### Aim of study

This study aimed to assess and analyse the impact of socio-demographic factors on maternal mortality in South Africa.

## Materials and methods

The same methodology previously used by this author was employed to process and prepare the data for this study, estimate the MMR and perform statistical analysis from the causes of death data [14]. This study was descriptive and retrospective and evaluated maternal deaths by socio-demographic characteristics from 2007 to 2015 in South Africa. Data were extracted from Causes of Death and Live Births datasets from StatsSA for the 2007–2015 years under review. StatsSA obtains copies of completed Death Notification Forms (DNFs) from the DHA for data processing and analysis of mortality and causes of death. Thereafter, data is processed in several stages: forms are sorted by year of deaths, labels of unique identifiers are pasted on each form, socio-demographic variables and causes of death are coded, data is captured, analysed and published in annual reports. Trained medical coders at StatsSA use ICD-10 for categories of causes of death to classify, ascertain and code the causes of death in accordance with the 10th Revision International Statistical Classification of Diseases and Related Health Problems (ICD-10) developed by the World Health Organization (WHO) [17, 18].

### Statistical analysis

Data for this study was converted from ASCII format to SPSS 25 (2017) and Software-R (2019) which were used for the analyses [19, 20]. All cases with the variable pregnant were selected from the nine causes of death data sets and new files were created and merged to form one file. The following variables were considered in the analysis: age in 5 year categories, province of death, place of death (hospital, home, other), occupation (professional, semi-professional, unspecified, non-professional), education (none, primary, high school, university, unspecified), marital status (unmarried, married/living together, unspecified), direct and indirect causes of death. Overall, several variables had a number of unspecified categories: 46.2% for education, 13.4% for marital status, 13.7% for occupation, 17.1% for place of death and 0.2% for province of death. Nevertheless, these variables were included in the analysis as they individually constituted less than 50% of missing values which is the maximum percentage statistically accepted for inclusion of missing values in statistical analyses [21]. Caution is advised when interpreting results for education, due to the huge under representation of the various categories in this variable.

The data for the 9 years was pooled first and subsequently broken down into triennia for further analyses and to get a more balanced MMR. The MMRs reported in this study (formula below) were calculated by dividing the recorded/estimated number of maternal deaths by the total recorded/estimated number of live births between 2007 and 2015 and multiplying the result by 100,000 [22, 23].

$$ \mathrm{Maternal}\ \mathrm{mortality}\ \mathrm{ratio}\ \left(\mathrm{MMR}\right)=\frac{\mathrm{Number}\ \mathrm{of}\ \mathrm{maternal}\ \mathrm{deaths}\ \mathrm{in}\ \mathrm{a}\ \mathrm{given}\ \mathrm{year}\ \mathrm{and}\ \mathrm{area}}{\mathrm{Number}\ \mathrm{of}\ \mathrm{live}\ \mathrm{births}\ \mathrm{in}\ \mathrm{the}\ \mathrm{same}\ \mathrm{year}\ \mathrm{and}\ \mathrm{area}}\times \mathrm{100,000}\ \mathrm{live}\ \mathrm{births} $$

The MMR was calculated by year, province and age. Frequencies were used to describe maternal deaths for each year, maternal characteristics, and all the independent variables relevant to the study. The final causes of death were grouped into 19 categories which were dichotomised and cross-tabulated with the independent variables to analyze how the causes differ in the different groups. The association between all independent variables by year, province, and cause-specific mortality was measured by cross tabulations and Chi-square tests and corresponding *P* values. Age group, province of death, place of death, occupational status, educational level, and marital status were used as the predictor variables/covariates. The risks of maternal death associated with each independent variable were presented by adjusted odds ratios (aORs) with corresponding 95% Confidence Interval (CI) using one covariate at a time and adjusting for all the other variables in one model.

## Results

The study population included 14,892 maternal deaths of women from 9 to 55 years of age who died between 2007 and 2015 and 10,687,687 live births (retrieved from StatsSA database) that took place during the same period.

**Fig. 1:**
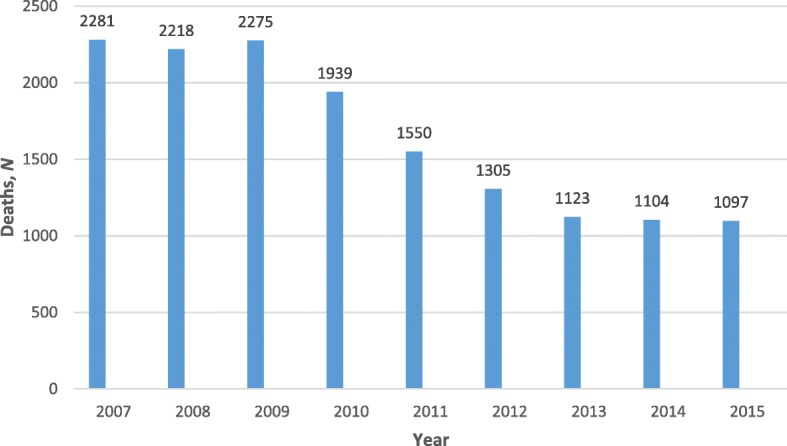
Maternal deaths reported to DHA: South Africa 2007 and 2015. Source: Statistics South Africa

**Fig. 2:**
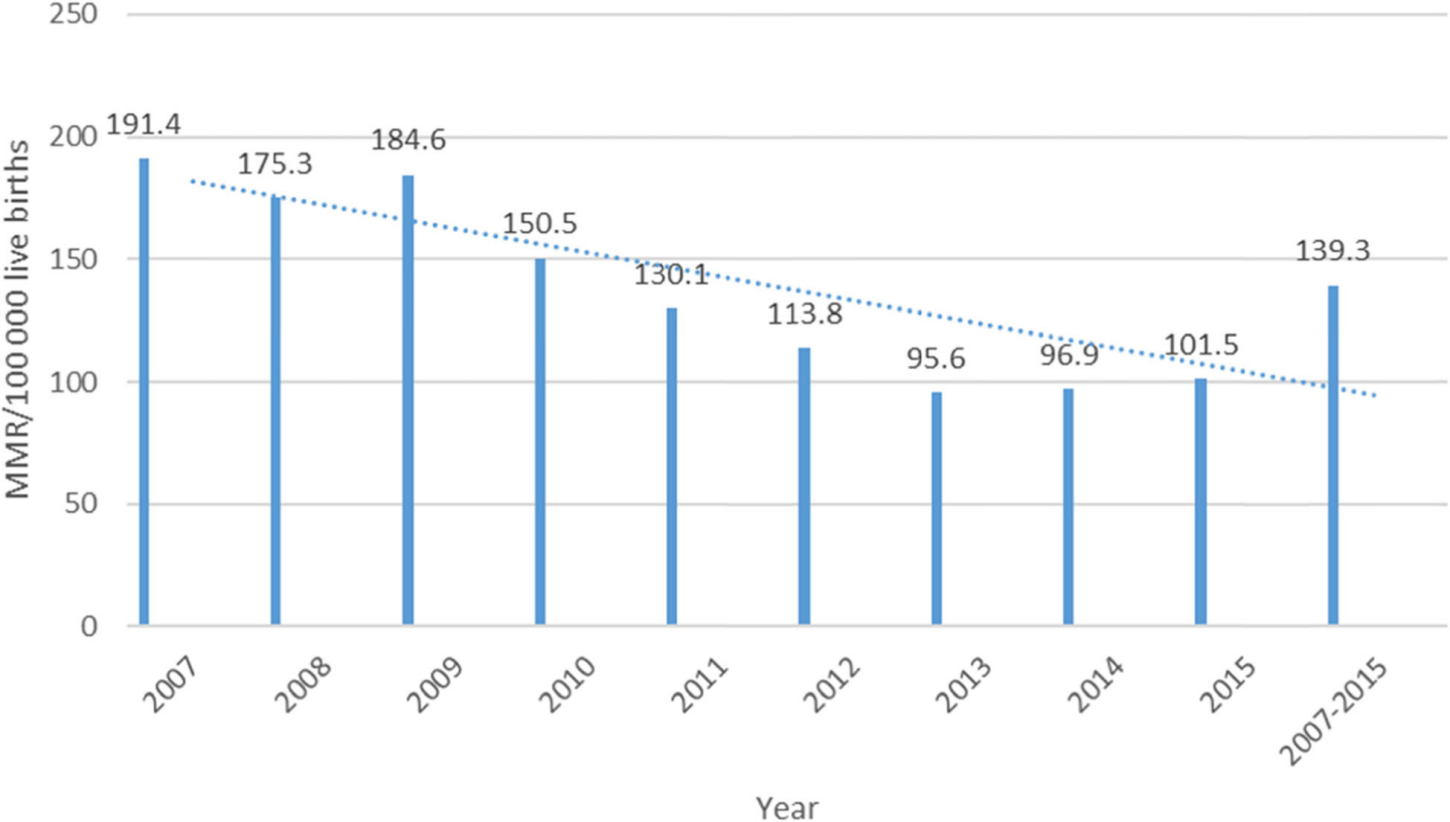
Maternal mortality ratio per 100 00 live births by year: South Africa 2007–2015. Source: computed from 2007 to 2015 maternal deaths and live births data. Statistics South Africa

**Fig. 3:**
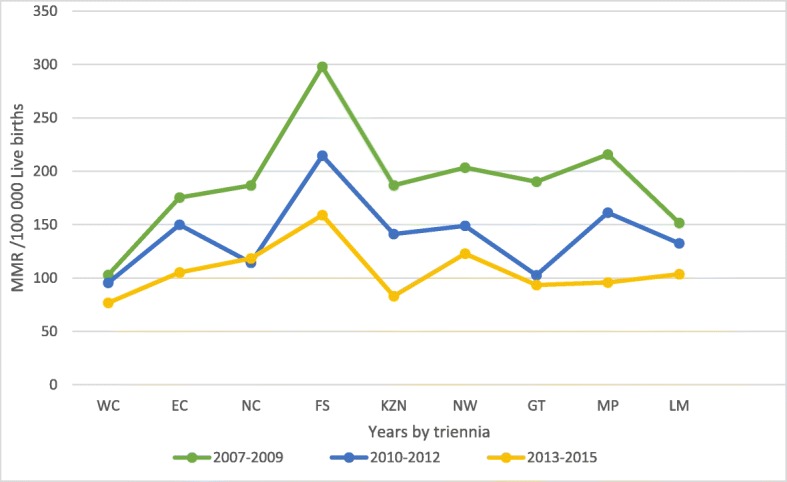
Maternal mortality ratio per 100,000 live births by province: South Africa 2007–2015

Largely, more maternal deaths occurred in 2007 (*n* = 2281) than in the other 8 years studied here. However, the year 2008 saw a slight decline followed by an incline in 2009 (2275) and thereafter a steady decline up to and including 2015 (Fig. 1). Over the years studied, the annual MMR ranged from 191.4/100000 live births in 2007 to 101.5/100000 live births in 2015, somewhat levelling off between 2013 and 2014. The corresponding ratio for the entire 2007–2015 period was 139.3/100000 live births (Fig. 2). Provincial level estimates in triennia (Fig. 3) indicate that the Free State (FS) had the highest MMR throughout this period much higher than the national average of 139/100000 live births while the Western Cape (WC) had the lowest MMR in the same period.

**Fig. 4:**
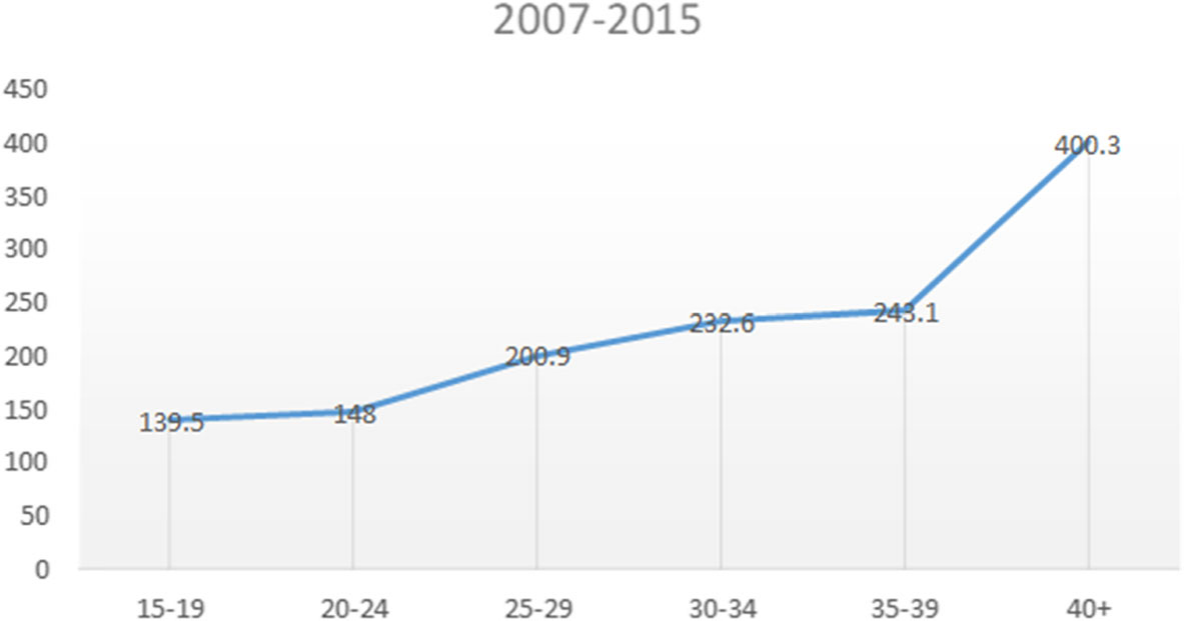
Maternal mortality ratio by age: South Africa 2007–2015

Furthermore, the MMR (139.3/100000 live births) during the 2007–2015 period is significantly lower than the 2002–2006 MMR (183/100000 live births) reported in a previous study by Bomela (2015) using 2002–2006 causes of death data from StatsSA, and Garenne et al. (2011) using data from the StatsSA 2007 Community Survey, demonstrating a noteworthy and impressive decline in MMR after 2006 [13, 14]. However, MMR increased with age throughout this period with the highest risk of dying reaching its peak among women aged 40 years or older, noticeably demonstrating how mortality increases with age (Fig. 4). The maternal mortality ratio starts low and rises steeply and non-linearly after age 30; the MMR curve becomes progressively steeper as age advances. Contrary to expectations, the age curve shows only a modest excess risk at ages 15–19 compared to ages 20–24. Similar findings were reported in the 2011–2013 NCCEMD report as well as in a study of 38 countries by the Population Council [10, 24].

The distribution of maternal deaths by province, age group, maternal education, marital status, occupation, place of death by province of death is presented in Table 1. As has been reported in other studies undertaken in South Africa [2–4], the Western Cape reported the second lowest number of deaths (6%) while Gauteng (GT) and KwaZulu-Natal (KZN) had the highest number of deaths, 21 and 22% respectively. Throughout this period deaths in KZN decreased steadily while deaths in GT although fluctuating were on the rise. The majority of women who died during this period were younger (mean age at death 31.1 years, (SD 37.2)), had an unspecified educational status, unmarried and died in a health care facility. Overall, a quarter (25.2%) of the deaths occurred in the 25 to 29 years age group. More than half (55.3%) of all the women who died during the study period were 29 years and younger, largely because those are the ages at which women are most likely to give birth and more deaths occur where there are more births [24].

**Table 1:** Sociodemographic characteristics of women with pregnancy related deaths by province: South Africa 2007–2015

Characteristic	WC	EC	NC	FS	KZN	NW	GT	MP	LP	OTHER	ALL
Maternal age											
10–14	4 (2.7)	31 (20.9)	5 (3.4)	12 (8.1)	37 (25.0)	10 (6.8)	23 (15.5)	12 (8.1)	13 (8.8)	1 (0.7)	148 (1.0)
15–19	80 (5.2)	244 (16.0)	35 (2.3)	120 (7.9)	397 (26.0)	99 (6.5)	254 (16.6)	129 (8.5)	167 (10.9)	1 (0.1)	1526 (10.2)
20–24	179 (5.9)	372 (12.3)	53 (1.8)	261 (8.6)	739 (24.4)	223 (7.4)	613 (20.3)	293 (9.7)	287 (9.5)	7 (0.2)	3027 (20.3)
25–29	256 (6.8)	460 (12.3)	86 (2.3)	273 (7.3)	819 (21.9)	285 (7.6)	834 (22.3)	332 (8.9)	395 (10.5)	7 (0.2)	3747 (25.2)
30–34	184 (5.8)	386 (12.2)	69 (2.2)	258 (8.1)	647 (20.4)	280 (8.8)	730 (23.0)	288 (9.1)	325 (10.2)	7 (0.2)	3174 (21.3)
35–39	115 (6.0)	223 (11.6)	35 (1.8)	149 (7.7)	357 (18.5)	163 (8.4)	470 (24.4)	174 (9.0)	236 (12.2)	8 (0.4)	1930 (13.0)
40+	91 (6.8)	192 (14.3)	41 (3.1)	131 (9.8)	240 (17.9)	116 (8.7)	259 (19.3)	122 (9.1)	144 (10.7)	4 (0.3)	1340 (9.0)
Maternal education											
None	15 (3.8)	51 (12.8)	20 (5.0)	28 (7.0)	90 (22.5)	40 (10.0)	46 (11.5)	55 (13.8)	52 (13.0)	3 (0.8)	400 (2.7)
Primary	48 (3.1)	255 (16.7)	52 (3.4)	192 (12.6)	354 (23.2)	133 (8.7)	139 (9.1)	190 (12.4)	160 (10.5)	5 (0.3)	1528 (10.3)
High School	110 (2.2)	682 (13.7)	105 (2.1)	517 (10.4)	1313 (26.3)	264 (5.3)	705 (14.1)	623 (12.5)	657 (13.2)	7 (0.1)	4983 (33.5)
University	19 (1.7)	117 (10.7)	20 (1.8)	99 (9.0)	281 (25.7)	54 (4.9)	214 (19.6)	133 (12.2)	153 (14.0)	4 (0.4)	1094 (7.3)
Unspecified	717 (10.4)	803 (11.7)	127 (1.8)	368 (5.3)	1198 (17.4)	685 (9.9)	2079 (30.2)	349 (5.1)	545 (7.9)	16 (0.2)	6887 (46.2)
Marital status											
Unmarried	490 (4.7)	1293 (12.4)	248 (2.4)	762 (7.3)	2648 (25.4)	878 (8.4)	1938 (18.6)	1025 (9.8)	1111 (10.7)	22 (0.2)	10,415 (69.9)
Married	176 (7.1)	401 (16.1)	40 (1.6)	277 (11.2)	332 (13.4)	158 (6.4)	539 (21.7)	234 (9.4)	318 (12.8)	8 (0.3)	2483 (16.7)
Unspecified	243 (12.2)	214 (10.7)	36 (1.8)	165 (8.3)	256 (12.8)	140 (7.0)	706 (35.4)	91 (4.6)	138 (6.9)	5 (0.3)	1994 (13.4)
Occupation											
Professional	19 (5.0)	49 (12.9)	5 (1.3)	37 (9.7)	76 (20.0)	15 (3.9)	88 (23.2)	47 (12.4)	43 (11.3)	1 (0.3)	380 (2.6)
Semi-professional	76 (6.7)	125 (11.0)	54 (4.8)	119 (10.5)	196 (17.3)	82 (7.2)	158 (13.9)	176 (15.5)	144 (12.7)	4 (0.4)	1134 (7.6)
Unspecified	97 (4.8)	250 (12.3)	38 (1.9)	167 (8.2)	519 (25.5)	158 (7.7)	448 (22.2)	182 (8.9	179 (8.8)	1 (0.0)	2039 (13.7)
Non-professional^a^	717 (6.3)	1484 (13.1)	227 (2.0)	881 (7.8)	2445 (21.6)	921 (8.1)	2489 (22.0)	945 (8.3)	1201 (10.6)	29 (0.3	11,339 (76.1
Death Place											
Hospital	579 (5.3)	1435 (13.2)	225 (2.1)	876 (8.1)	2537 (23.4)	767 (7.1)	2291 (21.1)	976 (9.0)	1155 (10.6)	18 (0.2	10,859 (72.9)
Home	52 (3.5)	168 (11.4)	34 (2.3)	198 (13.4)	295 (19.9)	168 (11.4)	186 (12.6)	222 (15.0)	150 (10.1)	7 (0.5)	1480 (9.9)
Unspecified	278 (10.9)	305 (11.9)	65 (2.5)	130 (5.1)	404 (15.8)	241 (9.4)	706 (27.7)	152 (6.0)	262 (10.3)	10 (0.4)	2553 (17.1)
Total	909 (6.1)	1908 (12.8)	324 (2.2)	1204 (8.1)	3236 (21.7)	1176 (7.9)	3183 (21.4)	1350 (9.1)	1567 (10.5)	35 (.2)	14,892 (100)

^a^This category is classified by StatsSA as inclusive of armed forces, occupation unspecified and not elsewhere classified

Most maternal deaths in the 10–24 years age group occurred in KZN (36.2%) while the majority of those in the 25+ age group occurred in GT (72%). The majority of deaths occurred among those whose educational status was unspecified (46.2%) and lowest among those with no education, primary school education and university education. Four provinces: FS, KZN, Mpumalanga (MP) and LP had higher numbers of deaths among those with a high school education than those whose educational status was unspecified. MP which is a rural province had the largest number in this group (46.1%). Almost 70% of maternal deaths during this period occurred amongst women who were unmarried, and the majority were from KZN (81.8%). More than three-quarters of deaths occurred among the non-professionals, while close to three quarters (72.9%) of the deaths occurred in a health care facility, and 9.9% of deaths occurred at home.

**Fig. 5:**
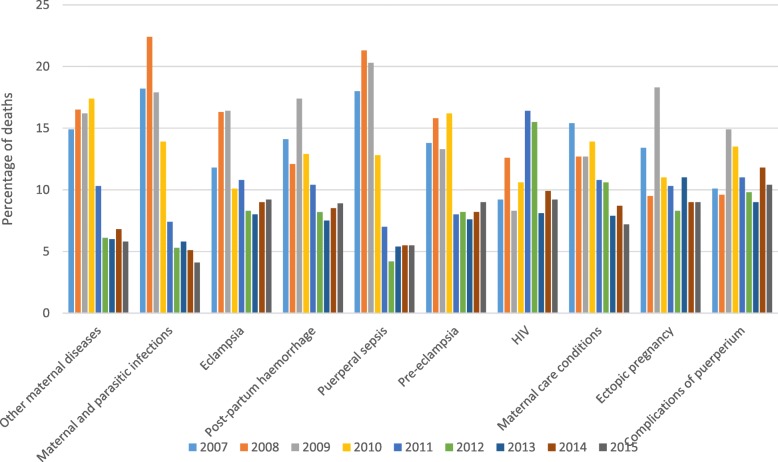
Cause-specific proportionate maternal mortality: South Africa 2007–2015

The proportionate maternal deaths by the top ten causes of death is illustrated in Fig. 5. In comparison to the 2007–2010 period a considerable decline in the majority of the top ten causes of death was noted between 2011 and 2015. The maternal infectious and parasitic diseases were the leading causes of death in 2007 (18.2%) and 2008 (22.4%), followed by puerperal sepsis in 2008 (21.3%) and 2009 (20.3%) and ectopic pregnancy (18.3%) in 2009. Other maternal disorders (17.4%) and pre-eclampsia (16.4%), were highest in 2010, while HIV was the leading cause of death in 2011 (16.4%) and 2012 (15.5%). In 2013 ectopic pregnancy (11%) was the leading cause of death, while complications of the puerperium were leading causes of death in 2014 (11.8%) and 2015 (10.4%).

The variations in the indirect and direct causes of death by socio-demographic characteristics are presented in Tables 2 and 3.

**Table 2:** Variations in the indirect causes of maternal deaths by socio-demographic characteristics: South Africa 2007–2015

Variables	Tuberculosis n (%)	HIV and related n (%)	Viral diseases n (%)	Immune system disorders n (%)	Other ill-defined n (%)	Accidental injury n (%)	Event of undetermined intent n (%)	Miscellaneous indirect n (%)
Age group (years)								
10–14	0 (0)	4 (0.4)	0 (0)	0 (0)	6 (2.2)	17 (3.7)	5 (2.6)	22 (1.6)
15–19	8 (3.0)	42 (4.3)	21 (4.7)	6 (1.8)	39 (14.0)	90 (19.7)	44 (22.7)	111 (8.2)
20–24	39 (14.4)	174 (18.0)	82 (18.4)	66 (20.0)	35 (12.5)	106 (23.2)	55 (28.4)	188 (14.0)
25–29	61 (22.5)	288 (29.8)	131 (29.4)	93 (28.2)	64 (22.9)	92 (20.2)	39 (20.1)	268 (19.9)
30–34	57 (21.0)	254 (26.2)	113 (25.3)	82 (24.8)	40 (14.3)	62 (13.6)	24 (12.4)	220 (16.3)
35–39	41 (15.1)	134 (13.9)	53 (11.9)	42 (12.7)	35 (12.5)	39 (8.6)	12 (6.2)	178 (13.2)
40+	65 (24.0)	71 (7.3)	46 (10.3)	41 (12.4)	60 (21.5)	50 (11.0)	15 (7.7)	359 (26.7)
Total	271(100)	967 (100)	446 (100)	330 (100)	279 (100)	456(100)	194 (100)	1346 (100)
Province of death								
Western Cape	14 (5.2)	111 (11.5)	25 (5.6)	11 (3.3)	34 (12.2)	52 (11.4)	16 (8.2)	114 (8.5)
Eastern Cape	40 (14.8)	115 (11.9)	68 (15.2)	35 (10.6)	33 (11.8)	60 (13.2)	31 (16.0)	169 (12.6)
Northern Cape	3 (1.1)	38 (3.9)	6 (1.3)	4 (1.2)	8 (2.9)	15 (3.3)	1 (0.5)	38 (2.8)
Free State	27 (10.0)	43 (4.4)	20 (4.5)	35 (10.6)	16 (5.7)	27 (5.9)	15 (7.7)	140 (10.4)
KwaZulu-Natal	86 (31.7)	304 (31.4)	116 (26.0)	61 (18.5)	50 (17.9)	110 (24.1)	38 (19.6)	263 (19.5)
North West	17 (6.3)	59 (6.1)	41 (9.2)	36 (10.9)	22 (7.9)	18 (3.9)	22 (11.3)	99 (7.4)
Gauteng	36 (13.3)	162 (16.8)	78 (17.5)	66 (20.0)	66 (23.7)	96 (21.1)	54 (27.8)	246 (18.3)
Other	1 (0)	0 (0)	1 (0.2)	2 (0.6)	1 (0.4)	1(0.2)	1 (0.5)	3 (0.2)
Total	271 (100)	967 (100)	446 (100)	330 (100)	279 (100)	456(100)	194 (100)	1346 (100)
Place of death								
Health care facility	201 (74.2)	835 (86.3)	384 (86.1)	268 (81.2)	73 (26.2)	123 (27.0)	65 (33.5)	831 (61.7)
Home	45 (16.6)	51 (5.3)	19 (4.3)	34 (10.3)	83 (29.7)	55 (12.1)	18 (9.3)	226 (16.8)
Other	25 (9.2)	81 (8.4)	43 (9.6)	28 (8.5)	123 (44.1)	278 (61.0)	111 (57.2)	289 (21.5)
Total	271 (100)	967 (100)	446 (100)	330 (100)	279 (100)	456(100)	194 (100)	1346 (100)
Occupation								
Professional	3 (1.1)	13 (1.3)	5 (1.1)	7 (2.1)	5 (1.8)	20 (4.4)	3 (1.5)	48 (3.6)
Semi-Professional	38 (14.0)	86 (8.9)	33 (7.4)	31 (9.4)	15 (5.4)	47 (10.3)	16 (8.2)	142 (10.5)
Unspecified	33 (12.2)	101 (10.4)	54 (12.1)	45 (13.6)	13 (4.7)	60 (13.2)	17 (8.8)	147 (10.9)
Non-professional^a^	197 (72.7)	767 (79.3)	354 (79.4)	247 (74.8)	246 (88.2)	329 (72.1)	158 (81.4)	1009 (75.0)
Total	271 (100)	967 (100)	446 (100)	330 (100)	279 (100)	456(100)	194 (100)	1346 (100)
Education								
None	15 (5.5)	27 (2.8)	9 (2.0)	11 (3.3)	11 (3.9)	9 (2.0)	5 (2.6)	68 (5.1)
Primary	42 (15.5)	91 (9.4)	57 (12.8)	41 (12.4)	26 (9.3)	42 (9.2)	20 (10.3)	170 (12.6)
High School	97 (35.8)	346 (35.8)	133 (29.8)	108 (32.7)	88 (31.5)	189 (41.4)	77 (39.7)	424 (31.5)
University	14 (5.2)	56 (5.8)	32 (7.2)	20 (6.1)	25 (9.0)	66 (14.5)	20 (10.3)	101 (7.5)
Unspecified	103 (38.0)	447 (46.2)	215 (48.2)	150 (45.5)	129 (46.2)	150 (32.9)	72 (37.1)	583 (43.3)
Total	271 (100)	967 (100)	446 (100)	330 (100)	279 (100)	456 (100)	194 (100)	1346 (100)
Marital Status								
Unmarried	204 (75.3)	704 (72.8)	326 (73.1)	249 (75.5)	190 (68.1)	341 (74.8)	149 (76.8)	926 (68.8)
Married	32 (11.8)	108 (11.2)	53 (11.9)	36 (10.9)	46 (16.5)	84 (18.4)	30 (15.5)	251 (18.6)
Unspecified	35 (12.9)	155 (16.0)	67 (15.0)	45 (13.6)	43 (15.4)	31 (6.8)	15 (7.7)	169 (12.6)
Total	271 (100)	967 (100)	446 (100)	330 (100)	279 (100)	456 (100)	194 (100)	1346 (100)

^a^This category is classified by StatsSA as inclusive of armed forces, occupation unspecified and not elsewhere classified

**Table 3:** Variations in the direct causes of maternal deaths by socio demographic characteristics: South Africa 2007–2015

Variables	Abortion n (%)	Hypertensive disorders n (%)	Other maternal disorders n (%)	Maternal care conditions n (%)	Complications of labour and delivery n (%)	Complications related to puerperium n (%)	Haemorrhage n (%)	Sepsis n (%)	Maternal infectious and parasitic diseases n (%)	Other ill defined	Miscellaneous direct n (%)
Age group (years)											
10–14	6 (0.7)	29 (1.6)	9 (1.6)	4 (0.9)	2 (0.4)	3 (0.5)	2 (0.3)	5 (0.8)	7 (0.4)	21 (0.9)	6 (2.3)
15–19	89 (10.0)	271 (15.0)	61 (11.00)	50 (11.3)	42 (9.0)	98 (16.4)	64 (10.0)	73 (12.2)	154 (7.9)	231 (9.6)	32 (12.3)
20–24	197 (22.1)	421 (23.4)	116 (20.9)	71 (16.0)	82 (17.5)	150 (25.0)	127 (19.9)	156 (26.2)	395 (20.4)	506 (21.0)	61 (23.5)
25–29	231 (25.9)	409 (22.7)	145 (26.1)	91 (20.5)	130 (27.8)	127 (21.2)	147 (23.1)	159 (26.7)	583 (30.1)	631 (26.2)	58 (22.3)
30–34	213 (23.9)	343 (19.0)	106 (19.1)	109 (24.6)	112 (23.9)	109 (18.2)	158 (24.8)	115 (19.3)	457 (23.6)	547 (22.7)	53 (20.4)
35–39	113 (12.7)	236 (13.1)	73 (13.1)	84 (19.0)	79 (16.9)	81 (13.5)	88 (13.8)	66 (11.1)	228 (11.8)	310 (12.9)	38 (14.6)
40+	43 (4.8)	92 (5.1)	46 (8.3)	34 (7.7)	21 (4.5)	31 (5.2)	51 (8.0)	22 (3.7)	115 (15.9)	166 (6.9)	12 (4.6)
Total	892 (100)	1801 (100)	556 (100)	443(100)	468 (100)	599(100)	637 (100)	596 (100)	1939 (100)	2412 (100)	260 (100)
Province of death											
Western Cape	46 (5.2)	100 (5.6)	27 (4.9)	13 (2.9)	22 (4.7)	53 (5.2)	14 (2.2)	8 (1.3)	100 (5.2)	115 (4.8)	34 (13.1)
Eastern Cape	98 (11.0)	248 (13.8)	70 (12.6)	60 (13.5)	70 (15.0)	66 (11.0)	96 (15.1)	97 (16.3)	248 (12.8)	286 (11.9)	18 (6.9)
Northern Cape	13 (1.5)	40 (2.2)	6 (1.1)	11 (2.5)	3 (0.6)	16 (2.7)	11 (1.7)	10 (1.7)	35 (1.8)	58 (2.4)	8 (3.1)
Free State	54 (6.1)	167 (9.3)	44 (7.9)	28 (6.3)	36 (7.7)	58 (9.7)	43 (6.8)	47 (7.9)	153 (7.9)	229 (9.5)	22 (8.5)
KwaZulu-Natal	201 (22.5)	304 (16.9)	136 (24.5)	90 (20.3)	89 (19.0)	81 (13.5)	121 (19.0	155 (26.0)	504 (26.0)	478 (19.8)	49 (18.8)
North West	54 (6.1)	159 (8.8)	44 (7.9)	38 (8.6)	38 (8.1)	46 (7.7)	59 (9.3)	47 (7.9)	144 (7.4)	208 (8.6)	25 (9.6)
Gauteng	227 (25.4)	380 (21.1)	103 (18.5)	92 (20.8)	111 (23.7)	196 (32.7)	141 (22.1)	104 (17.4)	395 (20.4)	562 (23.3)	68 (26.2)
Mpumalanga	85 (9.5)	176 (9.8)	50 (9.0)	42 (9.5)	32 (6.8)	37 (6.2)	69 (10.8)	61 (10.2)	188 (9.7)	253 (10.5)	16 (6.2)
Limpopo	109 (12.2)	224 (12.4)	75 (13.5)	66 (14.9)	66 (14.1)	44 (7.3)	81 (12.7)	65 (10.9)	170 (8.8)	219 (9.1)	20 (7.7)
Other	5 (0.6)	3 (0.2)	1 (0.2)	3(0.7)	1 (0.2)	2 (0.3)	2 (0.3)	2 (0.3)	2 (0.1)	4 (0.2)	0 (0)
Total	892 (100)	1801 (100)	556 (100)	443(100)	468 (100)	599(100)	637 (100)	596 (100)	1939 (100)	2412 (100)	260 (100)
Place of death											
Health care facility	603 (67.6)	1360 (75.5)	455 (81.8)	347 (78.3)	339 (72.4)	470 (78.5)	457 (71.7)	463 (77.7)	1641 (84.6)	1844 (84.6)	100 (38.5)
Home	83 (9.3)	138 (7.7)	30 (5.4)	23 (5.2)	26 (5.6)	46 (7.7)	60 (9.4)	77 (12.9)	125 (6.4)	273 (11.3)	68 (26.2)
Other	206 (23.1)	303 (16.8)	71 (12.8)	73 (16.5)	103 (22.0)	83 (13.9)	120 (18.8)	56 (9.4)	173 (8.9)	295 (12.2)	92 (35.4)
Total	892 (100)	1801 (100)	556 (100)	443(100)	468 (100)	599(100)	637 (100)	596 (100)	1939 (100)	2412 (100)	260 (100)
Occupation											
Professional	21 (2.4)	41 (2.3)	25 (4.5)	13 (2.9)	17 (3.6)	18 (3.0)	10 (1.6)	7 (1.2)	38 (2.0)	83 (3.4)	3 (1.2)
Semi-Professional	67 (7.5)	105 (5.8)	37 (6.7)	26 (5.9)	34 (7.3)	33 (5.5)	37 (5.8)	38 (6.4)	160 (8.3)	162 (6.7)	27 (10.4)
Unspecified	78 (8.7)	256 (14.2)	69 (12.4)	54 (12.2)	62 (13.2)	66 (11.0)	73 (11.5)	117 (19.6	396 (20.4)	364 (15.1)	34 (13.2)
Non-professional^a^	726 (81.4)	1399 (77.7)	425 (76.4)	350 (79.0)	355 (75.9)	482 (80.5)	517 (81.2)	434 (72.8)	1345 (69.4)	1803 (74.8)	196 (75.4)
Total	892 (100)	1801 (100)	556 (100)	443 (100)	468 (100)	599 (100)	637 (100)	596 (100)	1939 (100)	2412 (100)	260 (100)
Education											
None	25 (2.8)	41 (2.3)	15 (2.7)	12 (2.7)	11 (2.4)	10 (1.7)	12 (1.9)	21 (3.5)	37 (1.9)	54 (2.2)	7 (2.7)
Primary	92 (10.3)	151 (8.4)	43 (7.7)	50 (11.3)	41 (8.8)	39 (6.5)	70 (11.0)	68 (11.4)	207 (10.7)	253 (10.5)	25 (9.6)
High School	313 (35.1)	584 (32.4)	183 (32.9)	157 (35.4)	162 (34.6)	165 (27.5)	213 (33.4)	200 (33.6)	669 (34.5)	782 (32.4)	93 (35.8)
University	72 (8.1)	136 (7.6)	47 (8.5)	34 (7.7)	50 (10.7)	52 (8.7)	46 (7.2)	28 (4.7)	89 (4.6)	183 (7.6)	23 (8.8)
Unspecified	390 (43.7)	889 (49.4)	268 (48.2)	190 (42.9)	204 (43.6)	333 (55.6)	296 (46.5)	279 (46.8)	937 (48.3)	1140 (47.3)	112 (43.1)
Total	892 (100)	1801 (100)	556 (100)	443 (100)	468 (100)	599(100)	637 (100)	596 (100)	1939 (100)	2412 (100)	260 (100)
Marital Status											
Unmarried	649 (72.8)	1216 (67.5)	370 (66.5)	297 (67.0)	317 (67.7)	383 (63.9)	425 (66.7)	446 (74.8)	1374 (70.9)	1666 (69.1)	183 (70.4)
Married	140 (15.7)	312 (17.3)	111 (20.0)	87 (19.6)	98 (20.9)	123 (20.5)	133 (20.9)	72 (12.1)	296 (15.3)	430 (17.8)	41 (15.8)
Unspecified	103 (11.5)	273 (15.2)	75 (13.5)	59 (13.3)	53 (11.3)	93 (15.5)	79 (12.4)	78 (13.1)	269 (13.9)	316 (13.1)	36 (13.8)
Total	892 (100)	1801 (100)	556 (100)	443 (100)	468 (100)	599(100)	637 (100)	596 (100)	1939 (100)	2412 (100)	260 (100)

^a^This category is classified by StatsSA as inclusive of armed forces, occupation unspecified and not elsewhere classified

There were slight differences in the indirect causes of death in relation to the various age group categories. HIV and related causes (30%), viral diseases (29.4%), immune system disorders (28.2%), and other ill-defined causes (22.9%) were highest in the 25–29 years age group. Excluding the miscellaneous indirect causes (31.3%), HIV and related causes (23%) was the leading cause of maternal mortality among the indirect causes. In contrast, accidental injury (23.2%) and event of undetermined intent (28.4%) mainly occurred in the 20–24 years age group. Tuberculosis (24%) and miscellaneous indirect causes (26.7%) were highest in the 40+ age group. Maternal mortality was highest for women who died in KZN in four indirect causes and miscellaneous indirect causes, while GT had the highest number of deaths in three indirect causes. Health care facilities had the highest number of deaths in four indirect causes.

The occupation variable (in the original dataset) had a category of armed forces which included occupations that could not be classified elsewhere making it difficult to analyze. There was a high number of deaths in all seven indirect causes and miscellaneous indirect causes among non-professional women. Similarly, those with unspecified educational status had the highest number of deaths in five of the seven indirect causes and miscellaneous indirect causes. Unmarried women had the highest number of deaths in all indirect causes and miscellaneous indirect causes.

Similar to the indirect causes slight differences were identified in the direct causes of death in relation to the various age group categories. Other ill-defined diseases (23%) were the leading cause of death among direct causes, followed by maternal infectious and parasitic diseases (17%) and hypertensive disorders (17%). Hypertensive disorders (23.4%), complications related to the puerperium (25%) and miscellaneous direct (23.5%) were highest in the 20–24 years age group. Conversely, abortion (25.9%), other maternal disorders (26.1%), complications of labour and delivery (27.8%), sepsis (26.7%), maternal infectious and parasitic diseases (30.1%) and other ill-defined diseases (26.2%) occurred mainly in the 25–29 years age group. Maternal care conditions (24.6%) and haemorrhage (24.8%) were the leading causes of death in the 30–34 years age group.

GT had the highest number of deaths from seven direct causes and miscellaneous direct causes. The majority of maternal deaths from direct causes occurred in a health care facility. The majority of professionals died from miscellaneous direct causes, while non-professionals had the highest number of deaths in all ten main direct causes of death. Maternal deaths among those with unspecified educational status were highest in all ten direct causes and miscellaneous direct causes closely followed by those who had a high school education (Table 3).

The adjusted Odds Ratios for the different groups with respect to the direct causes of death are presented in Table 4. Although not statistically significant, the risk of dying from complications of labour and haemorrhage was high in all age groups and significantly high in all age groups for maternal infectious and parasitic diseases compared to the 9–14 year olds. Additionally, the risk of dying from complications of labour (aOR = 2.9 95% CI = 0.9–18.2) and maternal care conditions (aOR = 1.5, 95% CI = 0.6–5.2) was highest among the 35–39 year olds compared to the 9–14 year-olds. The 30–34 year olds had a higher risk of dying from abortion (aOR = 1.6, 95% CI = 0.7–4.2) and haemorrhage (aOR = 3.6, 95% CI = 1.1–22.3) compared to the 9–14 year olds. The 25–29 year-olds had a significantly higher risk of dying from maternal infectious and parasitic diseases (aOR = 4.1, 95% CI = 2.0–9.9) in comparison with the 9–14 year olds. The 20–24 year-olds had a higher risk of dying from sepsis (aOR = 1.8, 95% CI = 0.6–5.4) in comparison with the 9–14 year olds. The 15–19 year-olds had a higher risk of dying from complications of the puerperium (aOR = 2.6, 95% CI = 0.9–11.0) in comparison with the 9–14 year olds.

**Table 4:** Adjusted Odds Ratio (OR) and 95% Confidence Interval (CI) of direct maternal mortality causes by socio-demographic characteristics

Variables	Abortion OR (95% CI)	Hypertensive disorders OR (95% CI)	Other maternal disorders OR (95% CI)	Maternal care conditions OR (95% CI)	Complications of labour OR (95% CI)	Complications related to puerperium OR (95% CI)	Haemorrhage OR (95% CI)	Sepsis OR (95% CI)	Maternal infectious and parasitic diseases OR (95% CI)	Other ill-defined OR (95% CI)	Miscellaneous direct OR (95% CI)
Age group (years)											
10–14	1(Ref)	1(Ref)	1(Ref)	1(Ref)	1(Ref)	1(Ref)	1(Ref)	1(Ref)	1(Ref)	1(Ref)	1(Ref)
15–19	1.3(0.6–3.5)	0.8(0.5–1.3)	0.5(0.2–1.2)	1.1(0.4–4.0)	2.0(0.6–12.6)	2.6(0.9–11.0)	3.0(0.9–19.0)	1.7(0.7–4.9)	2.5(1.2–6.1)	1.0(0.6–1.7)	0.5(0.2–1.5)
20–24	1.5(0.7–4.0)	0.6(0.4–0.9)	0.5(0.2–1.1)	0.8(0.3–2.8)	2.0(0.6–12.3)	1.8(0.7–7.7)	3.1(0.9–19.1)	1.8(0.8–5.4)	3.3(1.6–7.9)	1.1(0.7–1.9)	0.5(0.2–1.4)
25–29	1.4(0.7–3.8)	0.4(0.3–0.7)	0.5(0.2–1.0)	0.8(0.3–2.9)	2.5(0.8–15.7)	1.1(0.4–4.8)	2.9(0.9–17.7)	1.5(0.7–4.5)	4.1(2.0–9.9)	1.1(0.7–1.9)	0.4(0.1–1.1)
30–34	1.6(0.7–4.2)	0.4(0.2–0.6)	0.4(0.2–0.9)	1.2(0.5–4.1)	2.5(0.8–15.8)	1.1(0.4–4.7)	3.6(1.1–22.3)	1.3(0.5–3.8)	3.7(1.8–9.0)	1.1(0.7–1.9)	0.4(0.2–1.2)
35–39	1.3(0.6–3.6)	0.5(0.3–0.7)	0.4(0.2–1.0)	1.5(0.6–5.2)	2.9(0.9–18.2)	1.3(0.5–5.7)	3.2(1.0–19.9)	1.2(0.5–3.7)	3.1(1.5–7.5)	1.0(0.6–1.8)	0.5(0.2–1.3)
40+	0.7(0.3–2.0)	0.2(0.1–0.4)	0.4(0.2–1.0)	0.9(0.3–3.2)	1.1(0.3–7.5)	0.7(0.2–3.3)	2.7(0.8–16.7)	0.5(0.2–1.7)	2.2(1.1–5.5)	0.7(0.4–1.3)	0.1(0.0–0.5)
Province of death											
Western Cape	1(Ref)	1(Ref)	1(Ref)	1(Ref)	1(Ref)	1(Ref)	1(Ref)	1(Ref)	1(Ref)	1(Ref)	1(Ref)
Eastern Cape	1.0(0.7–1.4)	1.2(0.9–1.6)	1.2(0.8–2.0)	2.1(1.1–4.0)	1.5(0.9–2.5)	0.5(0.4–0.8)	3.4(2.0–6.4)	5.9(3.0–13.3)	1.1(0.9–1.5)	1.1(0.9–1.4)	0.2(0.1–0.4)
Northern Cape	0.7(0.3–1.4)	1.2(0.8–1.8)	0.6(0.2–1.5)	2.4(1.0–5.5)	0.3(0.0–1.1)	0.9(0.5–1.6)	2.4(1.0–5.4)	3.5(1.3–9.4)	0.9(0.6–1.4)	1.5(1.0–2.1)	0.6(0.2–1.2)
Free State	0.9(0.5–1.3)	1.4(1.0–1.8)	1.3(0.8–2.2)	1.5(0.8–3.2)	1.2(0.7–2.2)	0.8(0.5–1.2)	2.4(1.3–4.7)	4.3(2.1–10.1)	1.1(0.8–1.5)	1.5(1.1–1.9)	0.4(0.2–0.7)
KwaZulu-Natal	1.2(0.8–1.7)	0.8(0.6–1.1)	1.5(0.9–2.3)	1.9(1.0–3.6)	1.1(0.7–1.8)	0.4(0.2–0.6)	2.6(1.5–4.9)	5.1(2.6–11.6)	1.4(1.1–1.7)	1.1(0.9–1.4)	0.3(0.2–0.6)
North West	0.8(0.5–1.3)	1.3(1.0–1.8)	1.3(0.8–2.3)	2.3(1.2–4.5)	1.3(0.8–2.4)	0.7(0.4–1.0)	3.4(1.9–6.5)	4.4(2.1–10.2)	1.1(0.8–1.5)	1.4(1.1–1.8)	0.5(0.2–0.8)
Gauteng	1.4(1.0–2.0)	1.1(0.8–1.4)	1.0(0.7–1.7)	1.9(1.1–3.6)	1.4(0.9–2.3)	1.0(0.7–1.4)	3.0(1.7–5.5)	3.7(1.9–8.3)	1.0(0.8–1.3)	1.4(1.1–1.7)	0.5(0.3–0.9)
Mpumalanga	1.2(0.8–1.8)	1.3(1.0–1.7)	1.3(0.8–2.2)	2.2(1.1–4.3)	0.9(0.5–1.7)	0.4(0.3–0.7)	3.7(2.1–7.0)	5.0(2.5–11.5)	1.2(0.9–1.6)	1.5(1.1–1.9)	0.2(0.1–0.4)
Limpopo	1.3(0.9–2.0)	1.4(1.1–1.8)	1.7(1.0–2.7)	2.8(1.5–5.4)	1.6(1.0–2.8)	0.4(0.3–0.7)	3.6(2.0–6.7)	4.8(2.4–11.1)	0.9(0.7–1.2)	1.0(0.8–1.3)	0.3(0.1–0.5)
Other	2.8(0.9–7.3)	0.8(0.2–2.4)	1.0(0.0–5.2)	6.4(1.4–21.3)	1.1(0.0–5.5)	1.0(0.1–3.7)	3.9(0.6–15.1)	7.2(1.0–30.8)	0.6(0.0–2.0)	0.8(0.2–2.3)	0.0(0.0–0.0)
Place of death											
Health care facility	1(Ref)	1(Ref)	1(Ref)	1(Ref)	1(Ref)	1(Ref)	1(Ref)	1(Ref)	1(Ref)	1(Ref)	1(Ref)
Home	1.0(0.8–1.3)	0.7(0.6–0.8)	0.4(0.3–0.6)	0.4(0.2–0.7)	0.5(0.3–0.8)	0.8(0.5–1.1)	0.9(0.7–1.2)	1.3(1.0–1.6)	0.5(0.4–0.6)	1.1(0.9–1.3)	5.5(4.0–7.7)
Other	1.5(1.2–1.7)	0.9(0.7–1.0)	0.6(0.5–0.8)	0.9(0.6–1.1)	1.3(1.0–1.6)	0.6(0.4–0.8)	1.1(0.9–1.4)	0.5(0.4–0.7)	0.4(0.3–0.5)	0.6(0.5–0.7)	3.6(2.7–4.8)
Occupation											
Non-professional^a^	1(Ref)	1(Ref)	1(Ref)	1(Ref)	1(Ref)	1(Ref)	1(Ref)	1(Ref)	1(Ref)	1(Ref)	1(Ref)
Professional	0.8(0.5–1.2)	0.9(0.6–1.2)	1.7(1.1–2.6)	1.0(0.5–1.7)	1.1(0.6–1.9)	1.1(0.6–1.8)	0.5(0.2–0.9)	0.5(0.2–1.1)	0.8(0.6–1.2)	1.4(1.1–1.8)	0.4(0–1.1.2)
Semi-professional	0.9(0.6–1.1)	0.8(0.6–1.0)	0.9(0.6–1.3)	0.6(0.4–1.0)	0.9(0.6–1.3)	0.8(0.6–1.2)	0.6(0.4–0.9)	0.9(0.6–1.3)	1.2(1.0–1.5)	0.8(0.7–1.0)	1.2(0.7–1.8)
Unspecified	0.5(0.4–0.7)	1.0(0.8–1.1)	0.8(0.6–1.1)	0.8(0.6–1.1)	1.0(0.7–1.3)	0.7(0.5–0.9)	0.7(0.6–1.0)	1.4(1.1–1.7)	1.7(1.5–1.9)	1.1(0.9–1.2)	0.9(0.6–1.4)
Education											
None	1(Ref)	1(Ref)	1(Ref)	1(Ref)	1(Ref)	1(Ref)	1(Ref)	1(Ref)	1(Ref)	1(Ref)	1(Ref)
Primary	0.9(0.6–1.5)	0.8(0.6–1.2)	0.7(0.4–1.3)	1.1(0.6–2.3)	0.9(0.4–1.9)	0.9(0.4–2.0)	1.5(0.8–3.1)	0.7(0.4–1.2)	1.4(0.9–2.1)	1.2(0.8–1.6)	0.7(0.3–2.0)
High School	0.9(0.5–1.4)	1.0(0.7–1.4)	0.9(0.5–1.6)	1.0(0.6–2.1)	1.0(0.5–2.0)	1.1(0.6–2.3)	1.4(0.8–2.7)	0.6(0.4–1.0)	1.3(0.9–1.9)	1.1(0.8–1.5)	0.9(0.4–2.2)
University	0.8(0.5–1.3)	1.0(0.7–1.6)	1.0(0.5–1.9)	1.0(0.5–2.1)	1.3(0.7–2.8)	1.5(0.8–3.3)	1.3(0.7–2.7)	0.4(0.2–0.7)	0.8(0.5–1.3)	1.2(0.8–1.6)	0.9(0.4–2.4)
Unspecified	0.8(0.5–1.2)	1.1(0.8–1.6)	1.0(0.6–1.8)	0.9(0.5–1.8)	0.9(0.5–1.8)	1.5(0.8–3.0)	1.4(0.8–2.8)	0.7(0.4–1.2)	1.4(1.0–2.1)	1.2(0.9–1.6)	0.6(0.3–1.6)
Marital status											
Unmarried	1(Ref)	1(Ref)	1(Ref)	1(Ref)	1(Ref)	1(Ref)	1(Ref)	1(Ref)	1(Ref)	1(Ref)	1(Ref)
Married	0.9(0.7–1.1)	1.2(1.0–1.4)	1.3(1.0–1.6)	1.1(0.8–1.4)	1.2(0.9–1.5)	1.5(1.2–1.9)	1.3(1.0–1.6)	0.8(0.6–1.0)	0.9(0.8–1.0)	1.1(0.9–1.2)	1.0(0.7–1.4)
Unspecified	0.8(0.6–1.0)	1.2(1.0–1.3)	1.1(0.8–1.4)	1.1(0.8–1.5)	0.8(0.6–1.1)	1.0(0.8–1.3)	0.9(0.7–1.2)	1.0(0.7–1.3)	1.0(0.9–1.2)	0.9(0.8–1.1)	0.9(0.6–1.4)

^a^This category is classified by StatsSA as inclusive of armed forces, occupation unspecified and not elsewhere classified

At provincial level, the risk of dying from haemorrhage and sepsis was significantly high in all the provinces compared to the women from the WC. Women from GT had a higher risk of dying from abortion-related causes (aOR = 1.4, 95% CI = 1.0–2.0) compared to the women from the WC. The risk of dying from hypertensive disorders (aOR = 1.4, 95% CI = 1.1–1.8), other maternal disorders (aOR = 1.7, 95% CI = 1.0–2.7), maternal care related conditions (aOR = 2.8, 95% CI = 1.5–5.4), and complications of labour (aOR = 1.6, 95% CI = 1.0–2.8) was significantly high for LP women compared to the women from the WC. Women from KZN had a significantly high risk of dying from maternal infectious and parasitic diseases (aOR = 1.4, 95% CI = 1.1–1.7) compared to women from the WC. Women from MP had a significantly higher risk of dying from haemorrhage (aOR = 3.7, 95% CI = 2.1–7.0) compared to the women from the WC. Eastern Cape (EC) women had a significantly higher risk of dying from sepsis (aOR = 5.9, 95% CI = 3.0–13.3) compared to women from the WC. Women from the FS had a significantly higher risk of dying from other ill-defined causes (aOR = 1.5, 95% CI = 1.1–1.9) compared to the women from the WC.

Women who died at home had a significantly higher risk of dying from miscellaneous direct causes (aOR = 5.5, 95% CI = 4.0–7.7) compared to those who died in a health care facility. Professional women had a significantly higher risk of dying from other maternal disorders (aOR = 1.7, 95% CI = 1.1–2.6) and other ill-defined causes (aOR = 1.4, 95% CI = 1.1–1.8) compared to those whose occupation was not classified. Married women had a significantly higher risk of dying from hypertensive disorders (aOR = 1.2, 95% CI = 1.0–1.4) other maternal disorders (aOR = 1.3, 95% CI = 1.0–1.6), complications of the puerperium (aOR = 1.5, 95% CI = 1.2–1.9), and haemorrhage (aOR = 1.3, 95% CI = 1.0–1.6) compared to unmarried women. Women with university education had a higher risk of dying from complications of labour (aOR = 1.3, 95% CI = 0.7–2.8) and complications of the puerperium (aOR = 1.5, 95% CI = 0.8–3.3) compared to women with no education. Women with primary school education had a higher risk of dying from haemorrhage (aOR = 1.5, 95% CI = 0.8–3.1) compared to women with no education.

The adjusted Odds Ratios for the different groups with respect to the indirect causes of death are shown in Table 5.

**Table 5:** Adjusted Odds Ratio (OR) and 95% Confidence Interval (CI) of indirect maternal mortality causes by socio-demographic characteristics

Variables	Tuberculosis OR (95% CI)	HIV and related OR (95% CI)	Viral diseases OR (95% CI)	Immune system disorders OR (95% CI)	Other ill-defined OR (95% CI)	Accidental injury OR (95% CI)	Event of undetermined intent OR (95% CI)	Miscellaneous indirect OR (95% CI)
Age group (years)								
10–14	–	1(Ref)	–	–	1(Ref)	1(Ref)	1(Ref)	1(Ref)
15–19	–	0.8(0.3–2.8)	–	–	0.5(0.2–1.5)	0.5(0.3–1.0)	1.0(0.4–3.2)	0.4(0.2–0.7)
20–24	–	1.9(0.7–6.2)	–	–	0.2(0.1–0.7)	0.3(0.1–0.6)	0.7(0.2–2.0)	0.3(0.2–0.6)
25–29	–	2.6(1.1–8.8)	–	–	0.4(0.1–1.1)	0.2(0.1–0.4)	0.3(0.1–1.2)	0.4(0.2–0.7)
30–34	–	2.9(1.2–9.6)	–	–	0.3(0.1–0.9)	0.1(0.1–0.3)	0.2(0.1–0.9)	0.4(0.2–0.7)
35–39	–	2.7(1.1–8.9)	–	–	0.4(0.1–1.2)	0.1(0.0–0.3)	0.2(0.0–0.7)	1.2(0.5–3.7)
40+	–	2.1(0.8–7.0)	–	–	0.9(0.4–2.6)	0.3(0.1–0.6)	0.3(0.1–1.2)	0.5(0.3–0.9)
Province of death								
Western Cape	1(Ref)	1(Ref)	1(Ref)	1(Ref)	1(Ref)	1(Ref)	1(Ref)	1(Ref)
Eastern Cape	1.2(0.6–2.4)	0.4(0.3–0.5)	1.3(0.8–2.1)	1.4(0.7–3.0)	0.4(0.2–0.7)	0.5(0.3–0.8)	0.9(0.5–1.9)	1.9(1.2–3.2)
Northern Cape	0.4(0.1–1.4)	0.8(0.5–1.3)	0.6(0.2–1.5)	0.9(0.2–2.7)	0.6(0.2–1.3)	0.7(0.3–1.2)	0.1(0.0–0.7)	0.6(0.5–0.8)
Free State	1.2(0.6–2.4)	0.2(0.1–0.3)	0.6(0.3–1.1)	2.2(1.1–4.7)	0.3(0.1–0.6)	0.3(0.2–0.5)	0.7(0.3–1.6)	0.8(0.5–1.2)
KwaZulu-Natal	1.5(0.8–2.9)	0.6(0.5–0.8)	1.2(0.8–2.0)	1.4(0.7–2.8)	0.4(0.3–0.7)	0.5(0.4–0.8)	0.7(0.3–1.3)	0.8(0.6–1.1)
North West	0.8(0.3–1.7)	0.3(0.2–0.5)	1.3(0.7–2.1)	2.4(1.2–5.0)	0.4(0.2–0.7)	0.2(0.1–0.4)	1.2(0.6–2.4)	0.6(0.5–0.8)
Gauteng	0.7(0.4–1.4)	0.3(0.2–0.4)	0.8(0.5–1.3)	1.6(0.9–3.3)	0.6(0.3–0.9)	0.5(0.3–0.7)	1.1(0.6–2.0)	0.5(0.4–0.8)
Mpumalanga	0.8(0.4–1.6)	0.3(0.2–0.5)	1.1(0.6–1.9)	2.4(1.2–5.1)	0.3(0.1–0.6)	0.4(0.2–0.6)	0.1(0.0–0.5)	0.6(0.4–0.7)
Limpopo	0.9(0.5–1.9)	0.2(0.1–0.3)	1.2(0.7–2.0)	1.8(0.9–3.9)	0.5(0.3–0.9)	0.4(0.2–0.6)	0.4(0.1–0.9)	0.8(0.6–1.0)
Other	1.4(0.8–7.9)	0.0(0.0–0.0)	1.2(0.0–6.0)	5.0(0.7–20.9)	0.4(0.0–2.3)	0.3(0.0–1.5)	1.2(0.0–7.0)	0.4(0.1–1.4)
Place of death								
Health care facility	1(Ref)	1(Ref)	1(Ref)	1(Ref)	1(Ref)	1(Ref)	1(Ref)	1(Ref)
Home	1.5(1.0–2.1)	0.4(0.3–0.5)	0.3(0.2–0.5)	0.8(0.5–1.1)	8.5(6.1–11.8)	3.2(2.2–4.4)	1.9(1.1–3.3)	1.7(1.4–2.0)
Other	0.5(0.3–0.8)	0.3(0.2–0.4)	0.4(0.3–0.6)	0.4(0.2–0.6)	6.7(4.9–9.0)	11.0(8.8–13.8)	7.3(5.3–10.1)	1.5(1.3–1.7)
Occupation								
Non-professional^a^	1(Ref)	1(Ref)	1(Ref)	1(Ref)	1(Ref)	1(Ref)	1(Ref)	1(Ref)
Professional	0.5(0.1–1.4)	0.4(0.2–0.8)	0.4(0.1–0.9)	0.9(0.4–1.9)	0.6(0.2–1.3)	1.8(1.0–2.9)	0.6(0.1–1.8)	1.4(1.0–1.9)
Semi-professional	1.8(1.2–2.6)	1.0(0.8–1.3)	1.0(0.6–1.4)	1.2(0.8–1.8)	0.4(0.2–0.8)	1.4(1.0–2.0)	1.2(0.6–2.0)	1.1(0.9–1.4)
Unspecified	0.8(0.5–1.2)	0.6(0.5–0.8)	0.8(0.6–1.0)	0.9(0.7–1.3)	0.3(0.1–0.5)	1.2(0.9–1.6)	0.6(0.3–1.0)	0.8(0.7–1.0)
Education								
None	1(Ref)	1(Ref)	1(Ref)	1(Ref)	1(Ref)	1(Ref)	1(Ref)	1(Ref)
Primary	0.6(0.3–1.2)	0.9(0.6–1.5)	1.7(0.9–3.8)	0.9(0.5–2.0)	0.7(0.3–1.5)	1.0(0.5–2.4)	0.9(0.3–2.8)	0.7(0.5–0.9)
High School	0.5(0.2–0.9)	1.1(0.7–1.8)	1.2(0.6–2.6)	0.8(0.4–1.6)	0.8(0.4–1.8)	1.4(0.7–3.0)	0.9(0.3–2.7)	0.6(0.5–0.9)
University	0.3(0.1–0.7)	0.9(0.5–1.5)	1.4(0.7–3.2)	0.7(0.3–1.5)	1.0(0.4–2.2)	1.8(0.9–4.1)	0.8(0.3–2.6)	0.7(0.5–1.0)
Unspecified	0.5(0.2–0.8)	0.9(0.6–1.4)	1.4(0.7–3.0)	0.8(0.4–1.7)	0.7(0.3–1.5)	0.7(0.3–1.5)	0.5(0.2–1.5)	0.6(0.4–0.8)
Marital status								
Unmarried	1(Ref)	1(Ref)	1(Ref)	1(Ref)	1(Ref)	1(Ref)	1(Ref)	1(Ref)
Married	0.6(0.4–0.9)	0.6(0.4–0.7)	0.7(0.5–0.9)	0.5(0.4–0.8)	0.9(0.6–1.2)	1.2(0.9–1.6)	1.1(0.7–1.7)	0.8(0.7–1.0)
Unspecified	1.1(0.7–1.6)	1.1(0.9–1.4)	1.1(0.8–1.5)	1.0(0.7–1.3)	1.0(0.7–1.5)	0.5(0.3–0.7)	0.5(0.3–0.9)	0.8(0.7–1.0)

μ: age for tuberculosis, viral diseases and immune system disorders was left out because there was not enough data to be included in the model for logistic regression analysis

^a^This category is classified by StatsSA as inclusive of armed forces, occupation unspecified and not elsewhere classified

Women in the 30–34 years age group had the highest risk of dying from HIV related causes (aOR = 2.9, 95% CI = 1.2–9.6) compared to the 9–14 year olds. Women from KZN had a higher risk of dying from tuberculosis (aOR = 1.5, 95% CI = 0.8–2.9) compared to WC women. The risk of dying from immune system disorders was significantly high for North West (NW) women (aOR = 2.4, 95% CI = 1.2–5.1) and the risk of dying from miscellaneous indirect causes was significantly high for EC women (aOR = 1.9, 95% CI = 1.2–3.2) compared to WC women. Women who died at home had a significantly higher risk of dying from tuberculosis (aOR = 1.5, 95% CI = 1.0–2.1), other ill-defined diseases (aOR = 8.5, 95% CI = 6.1–11.8), accidental injury (aOR = 3.2, 95% CI = 2.2–4.4), event of undetermined intent (aOR = 1.9, 95% CI = 1.1–3.3) and miscellaneous indirect causes (aOR = 1.7, 95% CI = 1.4–2.0) compared to women who died in a health care facility. Professional women had a significantly high risk of dying from accidental injury (aOR = 1.8, 95% CI = 1.0–2.9) and miscellaneous indirect causes (aOR = 1.4, 95% CI = 1.0–1.9) compared to non-professional women. The risk of dying from tuberculosis was significantly high for semi-professional women (aOR = 1.8, 95% CI = 1.2–2.6) compared to non-professional women. The risk of dying from viral diseases was high (aOR = 1.7, CI = 0.9–3.8) for women with primary schooling compared to those with no education. Women with a university education had a higher risk of dying from accidental injury (aOR = 1.8, 95% CI = 0.9–4.1) compared to those with no education.

## Discussion

Variations and uncertainties in MMR in South Africa are well-documented and recognized [25–28]. The RMS 2018 report [4] published estimates of MMRs and pregnancy-related mortality ratios (PRMRs) from various sources that were quite similar although produced from different data sources. Furthermore, the RMS report highlights the similarities in the incline and decline in MMR from these various sources. The current study found similar results to the RMS estimates. NCCEMD reports [6–11] published prior to the 2018 report have shown an increase in both the numbers and mortality ratios of iMMR in South Africa with the iMMR reaching a peak of 189 deaths per 100,000 live births in 2009. However, the 2018 report shows that since 2010 the iMMR declined substantially to 135 deaths per 100,000 live births in 2016 although that is still a long way to reach the SDG 3.1 global target of 70 deaths per 100,000 li0ve births goal by 2030 [1]. A similar trend to the above figures has been seen in this study where there was an incline in maternal deaths and MMR between 2007 and 2009 and both declined significantly after this period. The MMR in this study declined substantially from a high of 191 deaths per 100,000 live births in 2007 to a low of 139 deaths per 100,000 live births in 2015. These estimates are similar to the findings of the NCCEMD indicated above although the NCCEMD does not capture maternal deaths that occur outside the health care facilities. This significant decline in the MMR during the period under study indicates great strides in the improvement of maternal health in order to meet SDG 3.1 [1]. Tlou (2018) suggests that the higher iMMR between 2007 and 2010 was probably driven by the HIV epidemic which is embedded in our infection category and disproportionately affects pregnant women in South Africa [12]. Furthermore, the 2018 NCCEMD report, advocates that the decline in both maternal deaths and iMMR are largely due to improvements in HIV treatment with the extensive provision of Antiretroviral drugs (ARVs) to pregnant women [2, 3]. While such improvement is impressive, some provinces had maternal mortality ratios at least twice as high as the national level in 2015. Different socio-economic, demographic and environmental features in the provinces may be responsible for the differences in the maternal deaths and MMR.

Numerous challenges that obstruct safe maternal health care have been identified by the National Department of Health of South Africa and are indicated in the NCCEMD 2018 report as needing immediate attention. The health care system in South Africa is generally beset with a critical shortage of doctors, nurses and community healthcare workers; health facilities are either in disrepair or ill-equipped, equipment damaged or unavailable together with dysfunctional emergency medical services especially inter-facility transport between and within provinces [2]. Moodley et al. (2018) reported that around 60% of all maternal deaths in South Africa between 2014 and 2016 could have been prevented were it not for poor quality of care [3].

Various strategies across all health care facilities in the country to address the above challenges and improve MMR have not yielded the expected results, as MMR remains very high. Of greatest concern is the persistently high MMR in the FS which has continued to have a higher than national average MMR during the 9 years covered in this study. According to a report by Treatment Action Campaign (TAC) (2018) the shortage of human resources coupled with a large number of vacancies that remain unfilled in the province is a major issue in the Free State health care centres [29]. Schoon et al. (2011) identified inter-facility transport as a contributing factor to adverse pregnancy problems in the FS [30]. Similar studies done in Botswana, Tanzania and Bangladesh identified lack of transport to health care facilities; distance to a health care clinic and; quality of care as factors contributing to high maternal mortality and high MMR [31–34]. To address this problem, improved emergency transport, training on the management of obstetric emergencies, improvement of antenatal care and review of service planning were introduced as far back as 2011 in FS, however MMR remains high [30]. The majority of the community in the province has access to antenatal services, however, giving birth before reaching these services remains a problem. Delivery might occur in formal labour wards for the majority of patients, but not all hospitals have the staffing profile to provide a 24-h comprehensive emergency obstetric care service [35]. Further investigations are necessary to identify and address the root cause of the high MMR in this province besides the challenges indicated above which affect not only the FS but the entire country.

The contrast between provinces with the lowest and highest MMR is indicative of how much remains to be done. More comprehensive case studies will be required to devise more suitable interventions in these provinces. Liang et al. (2018) found similar results in their estimation of maternal mortality ratios in 2852 counties in China where some counties and provinces had higher than national average MMR despite extensive interventions directed at addressing the problem [36].

Provincial disparities persisted during 2007–2015 similar to what was reported in the 2002–2006 study by this author [14]. It should however be noted that there was a general decline of maternal mortality in all the provinces during these years. Gauteng (GT), which is the most populous and richest province with a 9.681 Gross Domestic product (GDP) per capita and KwaZulu-Natal (KZN) the second most populous province with third lowest GDP per capita (4.767) had the highest number of deaths, 21 and 22% respectively. The Western Cape which is the third most populous province and second richest province reported the second lowest number of deaths (6%.). WC has one of the best performing regional economies in South Africa, and is counted amongst the country’s best educational outcomes and health indicators. All the provinces in the country in various degrees experience similar challenges although there are variations between them. The unemployment rate is still unacceptably high and economy is under pressure, poverty and inequality is still rife, many households are not food secure, child and maternal death rates are still too high, education outcomes are still not at desired levels [37–39]. In comparison to the richer provinces the poorer provinces only have what are designated as Level 1 and Level 2 institutions (Community health centres and district hospitals, and Regional Hospitals respectively). In contrast, the richer provinces have Level 3 institutions (provincial tertiary and national central hospitals) which are better equipped with state of the art facilities. At present, provincial tertiary hospitals see a lot of cases from lower levels of care that die in their facilities due to either late referral or poor quality of care [3, 10].

WHO (2015) reported a significant decline in maternal mortality in developing countries [40]. This study confirms this decline in South Africa. Maternal deaths have steadily declined in South Africa since 2009; from a high of 2275 deaths to a low of 1097 deaths in 2015. As reported elsewhere in this paper the decline is largely due to improvements in HIV treatment with the extensive provision of ARVs to pregnant women. The decline in HIV is a significant contribution towards SDG 3.3 in the fight against the AIDS epidemic [1]. Furthermore, South Africa continues to improve its policies for further reduction of maternal deaths and MMR by establishing and implementing strategies to improve maternal health [3].

Although the MMR decreased during this period, it however increased with maternal age. A study recently undertaken in Central Gujarat, India (2019) supports these results. The study reported that the largest number of maternal deaths (83.1%) occurred in the age group of 20–34 but the MMR increased significantly in women 35 years old and above [41]. These results confirm what has been found in studies carried out in the United States and a WHO multi-country Survey on Maternal and Newborn Health on pregnancy-related mortality that advanced maternal age predisposes women to adverse pregnancy outcomes [42, 43]. The current study reported that the majority of women who died in South Africa between 2007 and 2015 were younger. The importance of providing contraception for teenagers and women over 34 years old in preventing unwanted pregnancies cannot be overstated and will greatly contribute towards achieving SDG 3.7 [1]. During the 2007–2015 period the contribution of the traditional causes of pregnancy related deaths (abortion, hypertensive disorders, other maternal disorders, maternal infectious and parasitic diseases and haemorrhage) increased significantly. A notable difference from the 2002–2006 study and the current study by the same author [14] is the steep rise in proportionate mortality due to hypertensive disorders from 9.1 to 17%; maternal infectious and parasitic diseases from 3.1 to 18.3%; viral diseases from 4.3 to 10.4%; HIV from 10.1 to 23% and; accidental injury from 4.9 to 10.6%. Several reports indicated earlier, show that an increasing number of pregnant women in South Africa have chronic health conditions such as hypertensive disorders, HIV, obesity and cardiac diseases which put pregnant women at risk of adverse outcomes. However, in this study most maternal deaths were attributed to direct obstetric causes mainly other maternal disorders, maternal infectious and parasitic diseases and hypertensive disorders.

The findings clearly show significant differences in pregnancy-related causes. Similar findings have been reported in other studies in Kenya [44] Angola [45], Nigeria [46], Bangladesh [47] and Pakistan [48]. It is disconcerting that a large number of women are still dying from pregnancy-related causes which can be diagnosed, controlled and treated during pregnancy. Women in the older age groups compared to their younger counterparts had the highest risk of dying from complications of labour, haemorrhage and maternal infectious and parasitic diseases. Statistics indicate that about two-thirds of maternal deaths in Africa are related to direct obstetric complications mainly haemorrhage, hypertension, sepsis, and obstructed labour [49]. Remarkably, the risk of dying from hypertensive disorders and other maternal disorders showed a decline (Table 4). Furthermore, the risk of dying from HIV-related causes, other ill-defined causes and accidental injury was reduced in all the provinces compared to the Western Cape (Table 5). There was also a higher risk of dying from maternal care conditions, haemorrhage and sepsis in all the provinces compared to the WC. Similar findings were reported in a WHO Systematic Analysis of global causes of maternal death which revealed that the above complications accounted for nearly 75% of all maternal deaths [50].

### Limitation of the study

The incomplete data on educational status, marital status, place of death, poor classification of occupational status and complete absence of data on the race/ethnicity variable was the biggest limitation of this study. Advanced statistical analyses on the impact of these variables on maternal mortality could not be performed. This lack of complete information on the death notification form (DNF) hinders accurate determination of the proportion of deaths that could have been classified under these variables [17]. Furthermore, the number of deaths in some of the variables tended to be high but were difficult to interpret since they may represent mixed groupings. South Africa even 25 years after the end of apartheid has been identified as the world’s most unequal society [51]. It is therefore important that researchers and policy-makers have supporting data that characterize racial disparities so that actions to reduce health inequalities can be planned accordingly.

## Conclusion

Regardless of the decline in maternal deaths and MMR the MMR is still too high in the provinces indicating that there are still a number of unaddressed challenges including the role of socio-demographic factors, and health care delivery. The persisting provincial variations indicate that more focus should be placed in improving quality of care at district, regional and provincial tertiary hospitals. This contrast requires cause-specific and target-specific intervention to arrest the high maternal mortality in the country. Improved quality of care in health care delivery will ensure adequate and immediate attention to not only maternal health problems but health care for the entire population at these institutions. In addition, there has to be a more concerted effort to address the poverty, economic and health inequality in South Africa to assist in improving health outcomes.

## Data Availability

The data that support the findings of this study are available from statistics South Africa but restrictions apply to the availability of these data, which were used under license for the current study, and so are not publicly available. Data are however available from the author upon reasonable request and with permission from statistics South Africa

## Abbreviations

ARVs: Antiretrovirals
DHA: Department of Home Affairs
DNF: Death Notification Form
EC: Eastern Cape
FS: Free State
GT: Gauteng
HIV: Human Immunodeficiency Virus
iMMR: Institutional Mortality Ratio
KZN: KwaZulu-Natal
LP: Limpopo Province
MMR: Maternal Mortality Ratio
MP: Mpumalanga
NC: Northern Cape
NCCEMD: National Committee on Confidential Enquiries into Maternal Deaths
NPR: National Population Register
NW: North West
PRMR: Pregnancy-related mortality ratio
RMS: Rapid Mortality Surveillance
SDG: Sustainable Development Goal
StatsSA: Statistics South Africa
WC: Western Cape
WHO: World Health Organization

## Footnotes

1 Maternal and late maternal deaths were defined as follows: Maternal death: the death of a woman while pregnant or within 42 days of termination of pregnancy, irrespective of the duration and site of the pregnancy, from any cause related to or aggravated by the pregnancy or its management but not from accidental or incidental causes. A late maternal death is the death of a woman from a direct or indirect obstetric cause more than 42 days but less than 1 year after termination [2]
